# On *α*-Limit Sets in Lorenz Maps

**DOI:** 10.3390/e23091153

**Published:** 2021-09-02

**Authors:** Łukasz Cholewa, Piotr Oprocha

**Affiliations:** 1 Faculty of Applied Mathematics, AGH University of Science and Technology, al. Mickiewicza 30, 30-059 Kraków, Poland; lcholewa94@gmail.com; 2 Centre of Excellence IT4Innovations, Institute for Research and Applications of Fuzzy Modeling, University of Ostrava, 30. dubna 22, 701 03 Ostrava, Czech Republic

**Keywords:** Lorenz map, renormalizable map, limit set, completely invariant set, entropy, 37E05, 37B05, 37E20

## Abstract

The aim of this paper is to show that α-limit sets in Lorenz maps do not have to be completely invariant. This highlights unexpected dynamical behavior in these maps, showing gaps existing in the literature. Similar result is obtained for unimodal maps on [0,1]. On the basis of provided examples, we also present how the performed study on the structure of α-limit sets is closely connected with the calculation of the topological entropy.

## 1. Introduction

Lorenz maps are interval maps which appear in a natural way as Poincaré sections in the Lorenz attractor. Their construction was independently discovered in works of Guckenheimer [1], Williams [2] and Afraimovich, Bykov and Shil‘nikov [3]. This is one of the possible tools that can be used to obtain a better insight into the widely studied Lorenz model. Families of Lorenz maps are usually derived from the so-called geometric Lorenz model, where, by definition, the Poincaré section leads to a map f:[0,1]→[0,1] satisfying the following three conditions:

1. There is a *critical point* c∈(0,1) such that *f* is continuous and strictly increasing on [0,c) and (c,1];
2. $\lim_{x\to c^{-}}f\left(x\right)=1$ and $\lim_{x\to c^{+}}f\left(x\right)=0$;
3. *f* is differentiable for all points not belonging to a finite set F⊆[0,1] and $\inf_{x\notin F}f^{\prime }\left(x\right)>1$.

Following the standard terminology, we call these maps *expanding Lorenz maps* due to uniform expansion provided by condition (3). The definition of the Lorenz map extends to maps defined on any compact interval [a,b] in an obvious way. Since the first papers, huge progress has been made towards understanding of the dynamics of Lorenz maps. A nice summary of different approaches and techniques (e.g., kneading theory of Milnor and Thurston, Markov partitions, renormalizations, etc.) can be found in the PhD thesis of M. St. Pierre, see [4] (cf. [5]) or the PhD thesis of B. Winckler, see [6] (cf. [7,8]). The simplest examples of Lorenz maps are transformations x↦βx+α. Even in this simple context, the dynamics is quite rich, and full characterization of standard notions as transitivity or mixing is quite challenging, e.g., see Glendinning [9], Glendinning and Sparrow [10] where a first insight into this topic has been gained and more recently in [11,12,13]. Beyond the linear case, a much more complex world of dynamics appears. The variety of examples increases even more if we drop the expanding assumption. It is possible to renormalize the dynamics an infinite number of times which leads to many interesting results, including strange attractors with irregular dynamical behavior (see [7] or [8]).

For the convenience of the reader, let us recall the definition of renormalization. Let f:[0,1]→[0,1] be an expanding Lorenz map. If there is a proper subinterval (u,v)∋c of (0,1) and integers l,r>1 such that the map g:[u,v]→[u,v] defined by

$$g\left(x\right)=\left\{\begin{array}{cc} f^{l}\left(x\right),\; & \mathrm{if}\;x\in \left[u,c\right),\\ f^{r}\left(x\right),\; & \mathrm{if}\;x\in (c,v], \end{array}\right.$$

is itself a Lorenz map, then we say that *f* is *renormalizable* or that *g* is a *renormalization* of *f*. The interval [u,v] is called the *renormalization interval*.

By expanding Assumption (3), in this paper, we encounter only finitely renormalizable Lorenz maps, that is, after some number of renormalizations, we obtain a Lorenz map which does not have renormalization. A nonwandering set of expanding Lorenz maps has been described in [14] with the following decomposition (see also [10]):

(1) $$\Omega \left(f\right)=\Omega_{0}\cup \ldots \cup \Omega_{n}\cup W$$

where $\Omega_{i}$ are invariant sets coming from consecutive nontrivial renormalizations, and *W* is the orbit of the interval *A* corresponding to the terminal renormalization, i.e., a renormalization which does not have any further renormalization.

A nonwandering set is tightly connected with the notion of ω-limit sets, which are among the most basic objects studied by the qualitative theory of dynamical systems. Blokh’s Decomposition Theorem (e.g., see [15]) provides full characterization of possible ω-limit sets in continuous interval maps, and [16] shows that the space of these sets is closed, in particular, a maximal ω-limit set always exists. These results do not apply directly to Lorenz maps (which are not continuous), but we can always present such a map by standard blow-up techniques, as maps on the Cantor set and view it as topological dynamical system. Then, knowledge from these two realms (interval dynamics and symbolic dynamics) can be used for the analysis. A dual concept to ω-limit sets are *α-limit sets*. In the case of homeomorphisms, they are simply ω-limit sets of the inverse map. In the case of non-invertible maps, the definition is not that simple nor obvious. We have at least three possible approaches. The first approach is to take the set of all accumulation points of the set of pre-images $f^{-n}\left(x\right)$ as an α-limit set, that is, the set

(2) $$\alpha \left(x\right)=\underset{n\geq 0}{⋂}\bar{\underset{k\geq n}{⋃}f^{-k}\left(\left\{x\right\}\right)}.$$

This approach is probably the most popular one. It appears in the work of Coven and Nitecki [17], who showed that for a continuous interval map, a point *x* is nonwandering if and only if x∈α(x), or, in a more recent paper, Cui and Ding [18] studied α-limit sets of unimodal interval maps. Another approach connects α-limits sets with single backward trajectories, e.g., see [19] for results of this approach in interval maps showing that all α-limits sets defined using backward trajectories are ω-limit sets but not conversely. Finally, [20] proposes to define the α-limit set as a union of limit sets calculated along all possible backward trajectories (so-called special α-limit sets). This way, a subset of α(x) is obtained, since it may happen that not all points in α(x) can be obtained as limits along the backward trajectory. Recent studies in [21,22] described basic properties of the special α-limit sets for interval maps. Depending on the approach, different properties can be guaranteed. For example, it is clear by the definition that α(x) is always a closed and invariant set (this is not the case of special α-limit sets, which are not necessarily closed as some examples show). In fact, the above mentioned studies show that accumulation points of backward trajectories behave similarly to accumulation points for forward trajectories only to some extent.

The main motivation for the present paper is Lemma 3.1 in [12], which is one of the main tools in the proofs of results in that paper. It asserts that if *f* is an expanding Lorenz map, then α(x) is a closed completely invariant set for every x∈[0,1]. Since, as we mentioned above, α(x) is always closed and invariant, the missing part is $f^{-1}\left(\alpha \left(x\right)\right)\subset \alpha \left(x\right)$. Let us remark here that when defining α(x) in [12], Ding considers sets $\left\{f^{-k}\left(x\right)\right\}$ which by definition consist of points *y* such that $\lim_{z\to y_{+}}f^{k}\left(z\right)=\lim_{z\to y_{-}}f^{k}\left(z\right)=x$. In our construction, we will consider a point *x* not belonging to the orbit of the critical point, so $\left\{f^{-k}\left(\left\{x\right\}\right)\right\}=f^{-k}\left(\left\{x\right\}\right)$ for every k≥0 in this case.

We show that the above mentioned statement of [12] is not true, by proving the following theorem:

**Theorem** **1.** *There exists an expanding Lorenz map f and x such that α(x) is not backward invariant, i.e., $f^{-1}\left(\alpha \left(x\right)\right)\setminus \alpha \left(x\right)\neq \emptyset$.*

In fact, the set α(x) in Theorem 1 will be one of the sets $\Omega_{i}$ in Equation (1). Let us also emphasize that Theorem 1 (and results of [12] in general, as explained below) have important consequences from the point of view of studies on structural complexity of Lorenz maps and their dynamics. Suppose *E* is a proper completely invariant closed set of an expanding Lorenz map *f*, put

$$e_{-}=\sup\left\{x\in E,x<c\right\},\;e_{+}=\inf\left\{x\in E,x>c\right\}$$

and

$$l=N\left(\left(e_{-},c\right)\right),\;r=N\left(\left(c,e_{+}\right)\right)$$

where N(U) is the smallest integer n≥0 such that $c\in f^{n}\left(U\right)$. Then, it follows from the results of [12], Theorem A (cf. [23]) that

$$f^{l}\left(e_{-}\right)=e_{-},\;f^{r}\left(e_{+}\right)=e_{+}$$

and the following map

(3) $$R_{E}f\left(x\right)=\left\{\begin{array}{cc} f^{l}\left(x\right), & x\in [f^{r}\left(c_{+}\right),c)\\ f^{r}\left(x\right), & x\in (c,f^{l}\left(c_{-}\right)] \end{array}\right.$$

is a renormalization of *f*. So, if α(x) was always backward invariant, it would define a renormalization when a proper subset of [0,1]. Unfortunately, as Theorem 1 shows, this is not always the case, and therefore, backward invariance needs additional checking. This comes with a surprise, since as we mentioned earlier, Lorenz maps are derived from the Lorenz model whose discretization is invertible (and smooth); thus, all α-limits sets are completely invariant. The problems arise when we consider dynamics induced on a Poincaré section, because the first return map is not defined at some points of the section which breaks the continuity and compactness. To make this map more accessible, reduction to the Lorenz map is made, but after this step, additionally, invertibility is lost. On the other hand, in a variety of α-limit sets backward invariance holds, see Section 7, and so for these sets (3) can be applied, provided that considered α-limit set is not [0,1].

Motivated by Theorem 1, we prove an analogous result for unimodal maps, that is, continuous maps f:[0,1]→[0,1] such that there exists a unique local maximum c∈(0,1), i.e., ${f|}_{\left[0,c\right)}$ is strictly increasing, ${f|}_{\left(c,1\right]}$ is strictly decreasing, and f(0)=f(1)=0.

**Theorem** **2.** *There exists a continuous unimodal map f on [0,1] and x such that α(x) is not backward invariant, i.e., $f^{-1}\left(\alpha \left(x\right)\right)\setminus \alpha \left(x\right)\neq \emptyset$.*

This result shows a gap in [18], Lemma 1(4), suggesting additionally that the proofs of the main results in [18] may be incomplete. In fact, by the same argument, we obtain that Theorem B(1) in [18] does not hold, see Remark 3.

The analysis of α-limit sets, nonwandering sets, and invariant sets in general, allows us to compute the (topological) entropy in constructed examples. Therefore, we use these examples as a testing ground to apply a few techniques to calculate the entropy of interval maps in concrete cases.

## 2. Symbolic Dynamics

**Figure 1:**
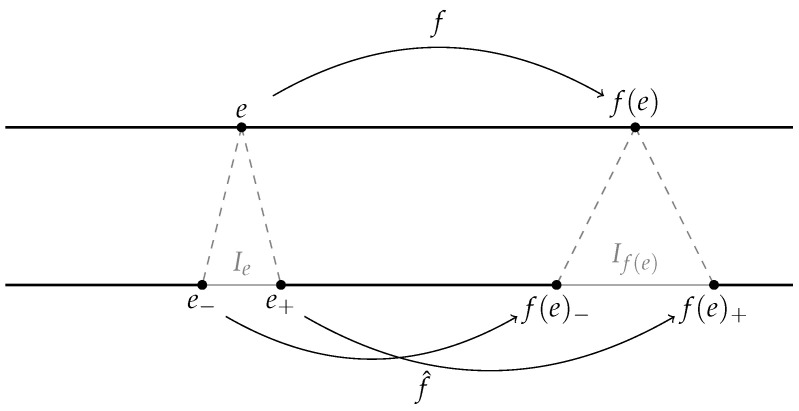
Illustration of “holes” $I_{e}$ and $I_{f(e)}$.

There is a standard technique to extend an expanding Lorenz map to a dynamical system acting on the Cantor set. Following [24], we change [0,1] into a Cantor set X, and *f* into a continuous map $\hat{f}$ by “doubling” the discontinuity point and its backward trajectory. Strictly speaking, all elements in $\left({⋃}_{n=0}^{\infty }f^{-n}\left(\left\{c\right\}\right)\right)\setminus \left\{0,1\right\}$ are doubled the same way as it is done in the standard Denjoy extension of rotation on the circle (e.g., see [25], Example 14.9).We easily see that this new space differs from the original interval [0,1] by, at most, countably many points and we do not modify endpoints; hence, clearly, the new space X is a Cantor set (we will provide an exact formula for the metric on X later). If we denote by $I_{e}$ a “hole” inserted in place of a point *e*, we may define $\hat{f}:X\to X$ by sending endpoints of $I_{e}$ to endpoints of $I_{f(e)}$, provided that the hole $I_{f(e)}$ is defined (see Figure 1).

In the case of e=c, we define the image of $I_{c}=\left[c_{-},c_{+}\right]$ by conditions imposed in the definition of the Lorenz map, that is, $\hat{f}\left(c_{-}\right)=1$ and $\hat{f}\left(c_{+}\right)=0$. Finally, if f(0)=c then we define $\hat{f}\left(0\right)=c_{+}$, and when f(1)=c we put $\hat{f}\left(1\right)=c_{-}$. The remaining case $f^{n}\left(0\right)=c$ (resp. $f^{n}\left(1\right)=c$) is dealt analogously, with the only difference that $\hat{f}\left(0\right)=a_{+}$ where $I_{f(0)}=\left[a_{-},a_{+}\right]$. Observe that in this case $I_{f(0)}$ is also the image of a complete hole, because f(1)>f(0). Reversing the above blow-up procedure, we obtain a map π:X→[0,1], which is clearly continuous.

To state a formal definition of the metric on X, we once again follow the standard approach described in [24]. We start by ordering elements in X referring to natural order in [0,1]. If x,y∈X are the endpoints of the same hole $I_{a}=\left[a_{-},a_{+}\right]$, then we define

$$x<y\Leftrightarrow x=a_{-}\;\mathrm{and}\;y=a_{+}.$$

For x,y∈X which are not bounding a single hole, the following is well defined:

x<y⇔π(x)<π(y).

For *x*, y∈X with x<y, we denote

$$n\left(x,y\right):=\min\left\{k\in N_{0}\;|\;\exists z\in \left(\overset{k}{\underset{j=0}{⋃}}f^{-j}\left(c\right)\right)\setminus \left\{0,1\right\}:x<z_{+}\;\mathrm{and}\;z_{-}<y\right\}.$$

Then, we introduce a metric on X by the formula

(4) $$d\left(x,y\right):=\left\{\begin{array}{cc} |\pi \left(x\right)-\pi \left(y\right)|+\frac{1}{N(x,y)+1}, & x\neq y\\ 0, & x=y \end{array}\right.,$$

where

$$N\left(x,y\right):=\left\{\begin{array}{cc} n(x,y); & x<y\\ n(y,x); & x>y \end{array}\right..$$

It is well known that the topology generated by the metric *d* coincides with the order topology on X. We have a natural partition of X by sets $P_{0}=\left[0,c_{-}\right]$ and $P_{1}=\left[c_{+},1\right]$. Denote $\Sigma_{2}={\left\{0,1\right\}}^{N_{0}}$ and let $\eta :X∋x\mapsto \eta \left(x\right)\in \Sigma_{2}$ be defined by $\eta {\left(x\right)}_{n}=a$ if ${\hat{f}}^{n}\left(x\right)\in P_{a}$. It is clear that η is a continuous map since $P_{0},P_{1}$ are closed and disjoint and $\hat{f}$ is continuous. The map η is also injective because if x<y, then by the expanding condition, there is an iteration *k* such that the images ${\hat{f}}^{k}\left(x\right)$, ${\hat{f}}^{k}\left(y\right)$ belong to different sets $P_{i}$ (this is also the case for points in the same hole, because each hole is eventually mapped onto $I_{c}$). By definition, η commutes between $\hat{f}$ and the shift map $\sigma :\Sigma_{2}\to \Sigma_{2}$ defined by $\sigma {\left(x\right)}_{n}=x_{n+1}$ for all n=0,1,…. In other words, $\left(X,\hat{f}\right)$ and (η(X),σ) are conjugate dynamical systems, that is, $\left(X,\hat{f}\right)$ is a subshift up to conjugacy.

The reader is referred to the books [26,27] for basic definitions, facts and constructions related to symbolic dynamics.

## 3. Construction of Expanding Lorenz Map f: Proof of Theorem 1

**Figure 2:**
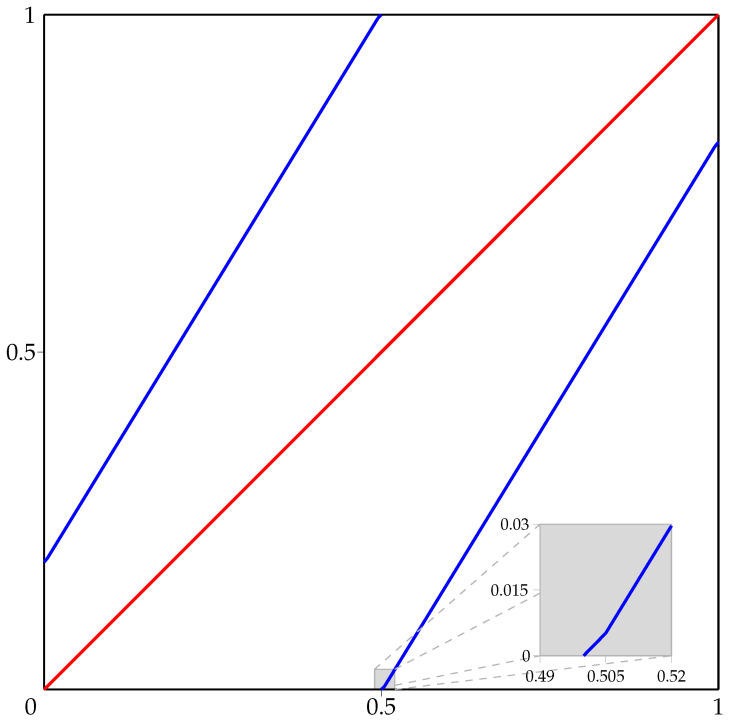
Graph of expanding Lorenz map *f* from Section 3.

The inspiration for our example comes from [10], Figure 2b. Among other interesting properties, an expanding Lorenz map whose kneading invariant is

(5) $$\left(k_{+},k_{-}\right)=\left(100{\left(011\right)}^{\infty },011{\left(100\right)}^{\infty }\right).$$

should have an invariant Cantor set and cannot have a constant slope. The reader not familiar with kneading sequences for Lorenz maps is referred to [10] and references therein.

One of the main goals of this section is to construct a map *f* with kneading invariant of the form (5).

At first, we will find parameters $\beta_{1},\beta_{2},\beta_{3}\in \left(1,+\infty \right)$ and $\alpha_{1},\alpha_{2}\in R$ such that

$$-1<p:=\frac{\alpha_{2}-\alpha_{1}}{\beta_{1}-\beta_{2}}<q:=\frac{1-\alpha_{2}}{\beta_{2}-\beta_{3}}<0$$

which ensures that the map g:[−1,1]→[−1,1] given by

(6) $$g\left(x\right)=\left\{\begin{array}{cc} \beta_{1}x+\alpha_{1}; & x\in [-1,p)\\ \beta_{2}x+\alpha_{2}; & x\in [p,q)\\ \beta_{3}x+1; & x\in [q,0)\\ \beta_{3}x-1; & x\in [0,-q]\\ \beta_{2}x-\alpha_{2}; & x\in (-q,-p]\\ \beta_{1}x-\alpha_{1}; & x\in (-p,1] \end{array}\right.,$$

is continuous. To ensure the appropriate form of $k_{+}$, we will require additionally that *g* satisfies the following conditions:

(7) $$\begin{array}{ccc} & & g(-1)\in [p,q),\\ & & g^{2}\left(-1\right)\in \left[q,0\right),\\ & & g^{3}\left(-1\right)\in \left(-p,1\right],\\ & & g^{4}\left(-1\right)\in \left(-q,-p\right],\\ & & g^{5}\left(-1\right)=g^{2}\left(-1\right). \end{array}$$

Our construction ensures that $\lim_{x\to p^{-}}g\left(x\right)=\lim_{x\to p^{+}}g\left(x\right)$ and $\lim_{x\to q^{-}}g\left(x\right)=\lim_{x\to q^{+}}g\left(x\right)$, therefore, *g* is continuous and strictly increasing on [−1,0). Note that for any map *g* of the form (6), we have the symmetry g(−x)=−g(x) for x∈[−1,1]∖{0}, which implies that *g* is also continuous and strictly increasing on (0,1] and the structure of $k_{-}$ is as desired. Moreover, $\lim_{x\to 0^{-}}g\left(x\right)=1$ and $\lim_{x\to 0^{+}}g\left(x\right)=-1$.

The conditions (7) together with formula (6) lead to the equality

$$g^{2}\left(-1\right)=\alpha_{2}+\left(\alpha_{1}-\beta_{1}\right)\beta_{2}=\left(\alpha_{2}+\left(\alpha_{1}-\beta_{1}\right)\beta_{2}\right)\cdot \left(\beta_{1}\beta_{2}\beta_{3}-1\right)=g^{5}\left(-1\right).$$

After simplification, we obtain the equation:

$$\left(\alpha_{2}+\left(\alpha_{1}-\beta_{1}\right)\beta_{2}\right)\cdot \left(\beta_{1}\beta_{2}\beta_{3}-2\right)=0,$$

which is satisfied for

$$\beta_{1}=\frac{6}{5},\;\beta_{2}=2\sqrt{\frac{2}{3}}\approx 1.63299,\;\beta_{3}=\frac{5}{2\sqrt{6}}\approx 1.02062$$

and

$$\alpha_{1}=\frac{2}{245}\cdot \left(132-25\sqrt{6}\right)\approx 0.57765,\;\alpha_{2}=\frac{2}{49}\cdot \left(32-3\sqrt{6}\right)\approx 1.00618.$$

Then, p≈−0.98969, q≈−0.01009 and simple calculations yield that the conditions (7) are fulfilled.

Next, let us denote $f\left(x\right)=\left(h^{-1}\circ g\circ h\right)\left(x\right)$, where h:[0,1]→[−1,1] is the affine map defined by h(x)=2x−1 and $h^{-1}:\left[-1,1\right]\to \left[0,1\right]$ is its inverse. Then, f:[0,1]→[0,1] is an expanding Lorenz map with critical point $c=\frac{1}{2}$. The graph of *f* is presented in Figure 2.

Direct calculations yield that

$$\begin{array}{ccc} & f\left(0\right)=\frac{1}{98}\cdot \left(43-10\sqrt{6}\right)\approx 0.18882, & f^{2}\left(0\right)=\frac{1}{98}\cdot \left(73-10\sqrt{6}\right)\approx 0.49495,\\ & f^{3}\left(0\right)=\frac{1}{98}\cdot \left(73+10\sqrt{6}\right)\approx 0.99484, & f^{4}\left(0\right)=\frac{1}{98}\cdot \left(25+22\sqrt{6}\right)\approx 0.80498,\\ & f\left(1\right)=\frac{5}{98}\cdot \left(11+2\sqrt{6}\right)\approx 0.81117, & f^{2}\left(1\right)=\frac{5}{98}\cdot \left(5+2\sqrt{6}\right)\approx 0.50505,\\ & f^{3}\left(1\right)=-\frac{5}{98}\cdot \left(-5+2\sqrt{6}\right)\approx 0.00515, & f^{4}\left(1\right)=\frac{1}{98}\cdot \left(73-22\sqrt{6}\right)\approx 0.19501 \end{array}$$

and $f^{2}\left(0\right)=f^{5}\left(0\right)$, $f^{2}\left(1\right)=f^{5}\left(1\right)$. This means that the kneading invariant of *f* is indeed given by Equation (5). Furthermore, observe that *f* has a 2-periodic orbit $O=\{z_{0},z_{1}\}$, where

$$z_{0}:=\frac{1}{490}\cdot \left(509-146\sqrt{6}\right)\approx 0.30892\;\mathrm{and}\;z_{1}:=\frac{1}{490}\cdot \left(-19+146\sqrt{6}\right)\approx 0.69107.$$

**Figure 3:**
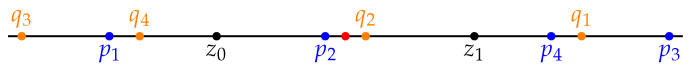
Relation between points $p_{i}$, $q_{i}$, $z_{i}$ and *c* for map *f* from Section 3.

Let us denote $p_{i}=f^{i}\left(0\right)$ and $q_{i}=f^{i}\left(1\right)$, whose ordering in [0,1] is depicted schematically in Figure 3. The critical point *c* is marked as red dot.

Consider the set

$$W=\left[0,q_{3}\right]\cup \left[p_{1},q_{4}\right]\cup \left[p_{2},q_{2}\right]\cup \left[p_{4},q_{1}\right]\cup \left[p_{3},1\right]$$

and observe that $f\left(\left[p_{2},q_{2}\right]\right)\subset \left[0,q_{3}\right]\cup \left[p_{3},1\right]$, therefore, f(W)⊂W. Since O∩W=∅, we have $f^{-k}\left(z_{0}\right)\cap W=\emptyset$ for every *k*. This implies that $\alpha \left(z_{0}\right)\subset I_{0}\cup I_{1}\cup I_{2}\cup I_{3}=\left[0,1\right]\setminus \mathrm{int}W$ where $I_{0}=\left[q_{3},p_{1}\right]$, $I_{1}=\left[q_{4},p_{2}\right]$, $I_{2}=\left[q_{2},p_{4}\right]$, $I_{3}=\left[q_{1},p_{3}\right]$. First, we are going to show that $\alpha (z_{0})$ is a Cantor set.

**Figure 4:**
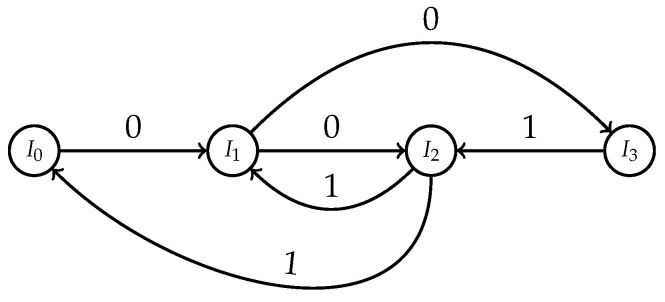
Sofic shift generated by sets $I_{0},I_{1},I_{2},I_{3}$.

Observe that $f\left(I_{0}\right)=I_{1}$, $f\left(I_{1}\right)⊃I_{2}\cup I_{3}$, $f\left(I_{2}\right)⊃I_{0}\cup I_{1}$ and $f\left(I_{3}\right)=I_{2}$. Consider the sofic shift Λ over the alphabet {0,1} whose graph representation is depicted in Figure 4.

Note that the kneading sequence of any point whose trajectory never leaves $Q=I_{0}\cup I_{1}\cup I_{2}\cup I_{3}$ is an element of Λ. Furthermore, since c∉Q and *f* is expanding, each point represents a unique element of Λ and each element has its representation. Therefore, we may conjugate the Cantor dynamical system (Λ,σ) with the maximal invariant set of *f* fully contained in *Q*. Furthermore, observe that by the covering relations between $I_{0},I_{1},I_{2},I_{3}$, for every word *w* allowed in the language of Λ, there exists a point z∈Q such that $f^{k}\left(z\right)=z_{0}$ for some k>0 and kneading sequence of *z* starts with *w*. This shows that $\alpha (z_{0})=\Lambda$. However, then, $f^{-1}\left(\alpha \left(z_{0}\right)\right)\setminus \alpha \left(z_{0}\right)=\left\{0,1\right\}$ because $p_{1},q_{1}\in \Lambda$. This concludes the proof of Theorem 1.

**Remark** **1.** *If we extend $\alpha (z_{0})$ to a completely invariant set, then we have to pass through c and, as a result, we obtain [0,1]. While f is renormalizable, by the results of [23], there is no proper, closed and completely invariant set that can define this renormalization in terms of Formula (3).*

**Remark** **2.** *It is clear that the map f is not topologically transitive, since in the case of transitive maps, the set $\cup_{n\geq 0}f^{-n}\left(\left\{x\right\}\right)$ is dense for every x∈[0,1] (e.g., see [23], Theorem 4.7).On the other hand, the map f does not have primary (n,k)-cycle (see terminology in [9]). This shows that characterization of transitivity by renormalizations and primary (n,k)-cycle developed in [9] (see also [13]) can only work for expanding Lorenz maps with constant slope.*

## 4. Decomposition of Nonwandering Set of f and Entropy

It is clear that the set Λ constructed in previous section satisfies Λ⊂Ω(f). In fact, $\Omega_{0}=\Lambda$ in Formula (1). It is a Cantor set, which is possible due to the fact that *f* is not of constant slope, neither is conjugate to an expanding Lorenz map of constant slope x↦α+βx(mod1) for any α,β. Namely, according to [10], Theorem 2 and [10], Section 6.1.2 (cf. [28]), sets $\Omega_{i}$ are always periodic orbits in the case of constant-slope expanding Lorenz maps.

Let us calculate the entropy of ${f|}_{\Omega_{0}}$, which is not hard, since Λ is sofic; so, we may use the well-known method based on the Frobenius–Perron theorem. If we consider a coincidence matrix related to the graph in Figure 4, then the leading eigenvalue is $\lambda =\frac{1}{2}\left(1+\sqrt{5}\right)=\frac{2}{\sqrt{5}-1}$. Therefore,

$$h_{top}\left(f{|}_{\Omega_{0}}\right)=h_{top}\left(\Lambda \right)=\log\lambda \approx 0.69424$$

where log here and later is always logarithm with base 2.

Let us also note that Λ is, in fact, a shift of finite type defined by the set of forbidden words F={000,111}. Since the associated shift of finite type is irreducible, the dynamics on $\Omega_{0}$ is transitive, and thus, we have an ergodic measure μ with entropy λ and support equal to $\Omega_{0}$. According to [14], entropy on sets $\Omega_{i}$ decreases with *i*, when nonzero, so $h_{top}\left(f\right)=\log\lambda$. Let us check that it is indeed the case here. We know that *f* has a terminal renormalization $F=(f^{3},f^{3})$ on $A=[p_{2},q_{2}]$. We already know that $f^{3}\left(p_{2}\right)=p_{2}$ and $f^{3}\left(q_{2}\right)=q_{2}$, so, up to linear change of slope, *F* represents a doubling map on the circle; hence, its entropy is $h_{top}\left(F\right)=\log2=1$. However, $h_{top}\left(f{|}_{W}\right)=\frac{1}{3}h_{top}\left(F\right)=\log\sqrt[{3}]{2}=1/3<\log\lambda$.

In fact, the above observed property of strong inequalities of entropies is a consequence of the general result that unimodal maps and symmetric Lorenz maps have a unique measure of maximal entropy (e.g., see [29,30], respectively; cf. [31], Corollary 3.7).

There is yet another method of calculating entropy of an expanding, finitely renormalizable Lorenz map, provided we know its kneading invariant (see [32,33,34], cf. [14]). Define power the series $k_{+}\left(t,t\right):=\sum_{i=0}^{\infty }a_{i}t^{i}$, where $a_{i}=1$ if the *i*-th symbol of the kneading invariant $k_{+}$ is 1 and $a_{i}=-1$ in the opposite case ($k_{-}\left(t,t\right)$ is defined the same way, using $k_{-}$). For the map *f*, using Equation (5) defining its kneading invariant, we obtain

$$\begin{array}{cc} k_{+}\left(t,t\right) & =1-t-t^{2}-t^{3}+t^{4}+t^{5}-t^{6}+t^{7}+t^{8}-\ldots \\ & =1-t-t^{2}+t^{3}\left(-1+t+t^{2}-t^{3}+t^{4}+t^{5}-\ldots \right)\\ & =1-t-t^{2}+t^{3}\left(k_{+}\left(t,t\right)-2+2t+2t^{2}\right), \end{array}$$

so

$$k_{+}\left(t,t\right)=\frac{1-t-t^{2}-2t^{3}+2t^{4}+2t^{5}}{1-t^{3}}$$

and by symmetry of the kneading invariant, $k_{-}\left(t,t\right)=-k_{+}\left(t,t\right)$. Therefore, we obtain

$$P_{f}\left(t,t\right):=k_{+}\left(t,t\right)-k_{-}\left(t,t\right)=2k_{+}\left(t,t\right)=2\cdot \frac{1-t-t^{2}-2t^{3}+2t^{4}+2t^{5}}{1-t^{3}}.$$

Easy calculations yield that $P_{f}\left(t,t\right)$ has two roots

$$t_{0}=\frac{1}{\sqrt[{3}]{2}}\approx 0.79370\;\mathrm{and}\;t_{1}=\frac{1}{2}\cdot \left(\sqrt{5}-1\right)\approx 0.61803$$

in the interval (0,1). By the results of [14], these roots correspond to entropies on the sets $\Omega_{i}$ for *i* where the entropy is positive. This method is not perfect because some of the zeros may not represent entropies. In the considered example, we have a 1-1 correspondence. The main difficulty with the described method that comes in practical applications is that we need a formal argument revealing what form the kneading invariant really has. Its numerical approximation may be not sufficient.

## 5. Unimodal Example: Proof of Theorem 2

**Figure 5:**
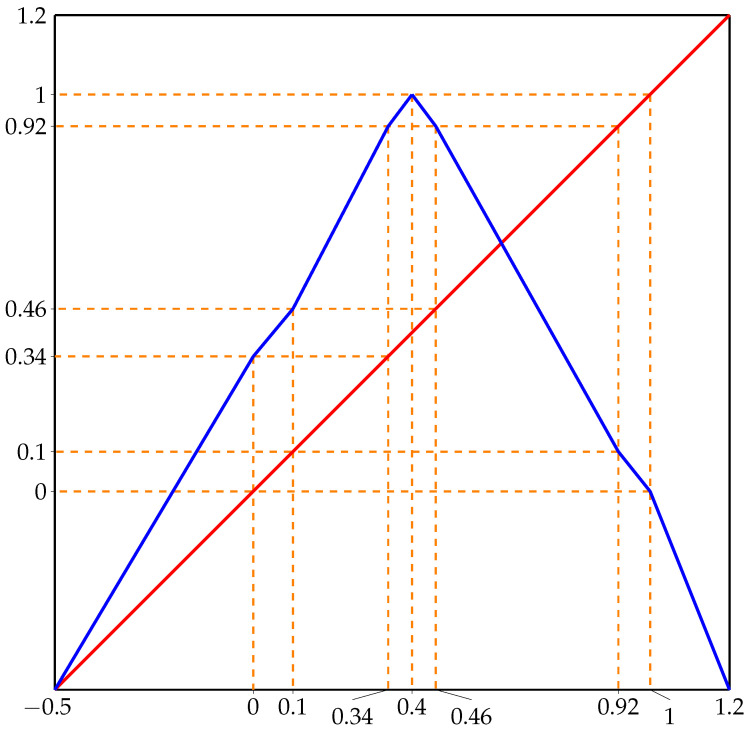
Graph of map *g* defined by Equation (8).

Let us consider a map g:[−0.5,1.2]→[−0.5,1.2] given by (see Figure 5):

(8) $$g\left(x\right)=\left\{\begin{array}{cc} 1.68x+0.34; & x\in [-0.5,0)\\ 1.2x+0.34; & x\in [0,0.1)\\ \frac{23}{12}x+\frac{161}{600}; & x\in [0.1,0.34)\\ \frac{4}{3}x+\frac{7}{15}; & x\in [0.34,0.4)\\ -\frac{4}{3}x+\frac{23}{15}; & x\in [0.4,0.46)\\ -\frac{41}{23}x+1.74; & x\in [0.46,0.92)\\ -1.25x+1.25; & x\in [0.92,1)\\ -2.5x+2.5; & x\in [1,1.2] \end{array}\right..$$

The initial map *g* is defined on interval [−0.5,1.2] because we want to arrange on [0,1] specific dynamical behavior, which makes its fynamics easier to study. Let $f\left(x\right)=\left(h^{-1}\circ g\circ h\right)\left(x\right)$, where h:[0,1]→[−0.5,1.2] is the map defined by h(x)=1.7x−0.5 and $h^{-1}:\left[-0.5,1.2\right]\to \left[0,1\right]$ is its inverse. Then, f:[0,1]→[0,1] is a unimodal map with the turning point $c=h^{-1}\left(0.4\right)\approx 0.52941$. Moreover, observe that *f* has 2-periodic orbit $O=\{x_{0},x_{1}\}$, where

$$x_{0}\approx 0.46215\;\mathrm{and}\;x_{1}\approx 0.77402.$$

**Figure 6:**
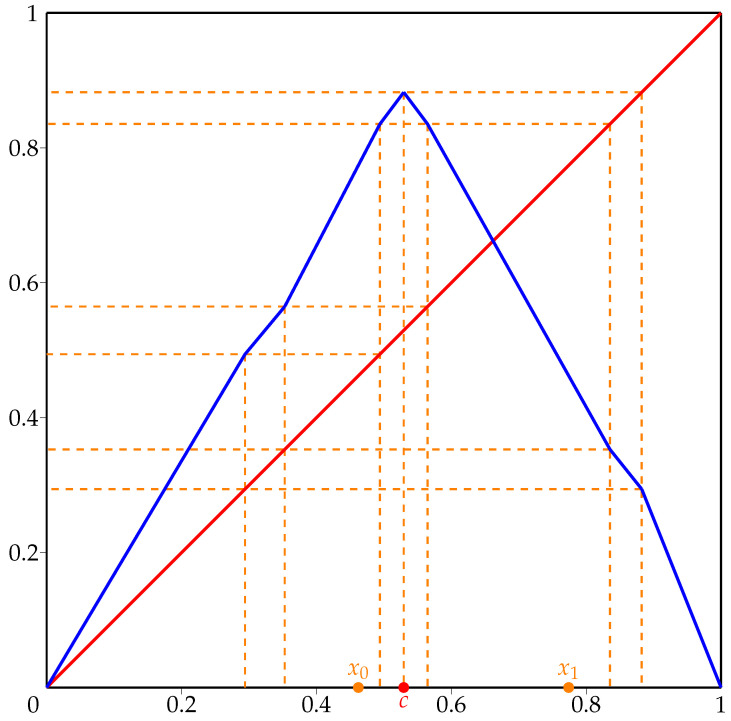
Graph of unimodal map *f* from Section 5.

The graph of *f* is presented in Figure 6. The points $x_{0}$ and $x_{1}$ are marked as orange dots.

From the formulas defining map *g*, it is easy to see that the set Q=[0,0.1]∪[0.34,0.46]∪[0.92,1] is invariant for *g* and intervals $I_{0}=\left[0.1,0.34\right]$ and $I_{1}=\left[0.46,0.92\right]$ satisfy $g\left(I_{0}\right)=I_{1}$, $g\left(I_{1}\right)⊃I_{0}\cup I_{1}$. Therefore, repeating the argument similar to the one in Section 3, we obtain that $I_{0},I_{1}$ contains a strongly invariant set Λ consisting exactly of points that never leave $I_{0},I_{1}$, ${g|}_{\Lambda }$ is transitive and conjugated to the shift of finite type $\Lambda \subset {\left\{0,1\right\}}^{N}$ defined by the forbidden word 00. However, the periodic point 0.1 of period 3 must then be an element of Λ; thus, it corresponds to periodic point ${\left(011\right)}^{\infty }$. Clearly, the turning point is eventually periodic finishing in this periodic orbit. Denote $\hat{\Lambda }=h^{-1}\left(\Lambda \right)$, then, clearly $f^{-n}\left(O\right)$ does not contain endpoints of the set $h^{-1}\left(I_{0}\cup I_{1}\right)$ since it cannot contain other periodic orbit. In particular, $\alpha \left(x_{0}\right)=\hat{\Lambda }$ but $c\notin \alpha (x_{0})$. On the other hand, $h^{-1}\left(1\right)\in \alpha \left(x_{0}\right)$ and so $c\in f^{-1}\left(\alpha \left(x_{0}\right)\right)$. This proves Theorem 2.

Let us finish this section by calculating the entropy of *f* which is the same as calculating the entropy of *g*. As we proved a moment ago, Λ contains all the recurrent points of *g* in $I_{0}\cup I_{1}$ and its entropy is equal to the entropy of the subshift obtained by the forbidden word 00. Then, the associated 2×2 coincidence matrix has the leading eigenvalue $\lambda =\frac{1}{2}\cdot \left(1+\sqrt{5}\right)\approx 1.61803$.

Next, observe that on the set *Q* we have a natural Markov partition [0,0.1], [0.34,0.4], [0.4,0.46], [0.92,1] and the associated Markov graph has the form:

$$\begin{array}{ccc} \left[0,0.1\right] & \to & [0.34,0.4],[0.4,0.46];\\ {}[0.34,0.4],[0.4,0.46] & \to & [0.92,1];\\ {}[0.92,1] & \to & [0,0.1]. \end{array}$$

This proves that $g^{3}$ keeps [0,0.1] invariant and is conjugated on this set with the standard tent map (unimodal map of constant slope 2). In particular, ${g|}_{Q}$ has entropy $\frac{1}{3}$, so recalling calculations in Section 4, we see that 1/3<logλ. However, outside of the interval [0,1], the function *g* has a unique fixed point (an endpoint) and the second endpoint is mapped onto it. All other points are eventually mapped into [0,1] which is *g*-invariant. This shows that

$$h_{\mathrm{top}}\left(f\right)=h_{\mathrm{top}}\left(g\right)\approx \log\left(1.61803\right)\approx 0.69424.$$

**Remark** **3.** *Observe that the argument from the proof of Theorem 2 can be repeated with any periodic point in $\hat{\Lambda }$ whose period is not 3 in place of orbit O. Note that f has the unique fixed point p∈(0,1). Clearly, $p\in \hat{\Lambda }$ since $1^{\infty }$ is the unique fixed point in the associated subshift. Therefore, we have $p\in \alpha \left(p\right)=\alpha \left(x_{0}\right)$ and also $f^{-1}\left(\alpha \left(p\right)\right)\setminus \alpha \left(p\right)\neq \emptyset$. On the other hand, it is not hard to see that $\alpha \left(p\right)=\bar{\cup_{n\geq 0}f^{-n}\left(\left\{p\right\}\right)}$. This contradicts [18], Theorem B(1), because for the unique fixed point p of f, the set $D:=\bar{\cup_{n\geq 0}f^{-n}\left(\left\{p\right\}\right)}$ is not backward invariant, contrary to the statement in [18].*

## 6. Continuous Piecewise Affine Maps

The aim of this section is to show that the construction in Section 3 cannot necessarily be extended to similar results for continuous maps. Strictly speaking, we will show that if we “fill” holes when extending the Lorenz map (i.e., extend Cantor set X to its convex hull), then the considered α-limit set will no longer satisfy $f^{-1}\left(\alpha \left(z_{0}\right)\right)\setminus \alpha \left(z_{0}\right)\neq \emptyset$.

**Figure 7:**
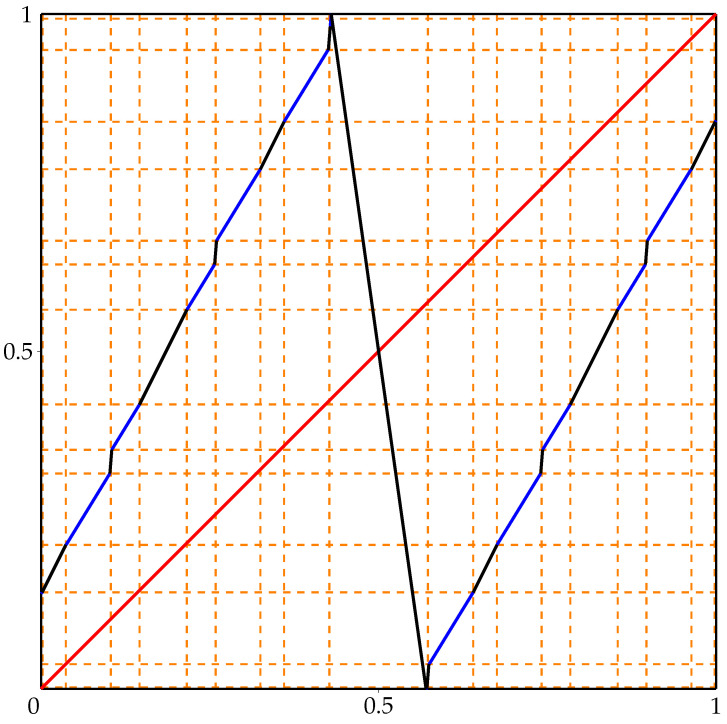
Graph of “blow up” of expanding Lorenz map leading to map *g* from Section 6. Parts of the graph over a few larger “filled holes” are marked in black.

To do so, we will analyze the properties of a piecewise affine map obtained by “filling” the holes in the Cantor set X induced by the Lorenz map from Section 3. Let us start by embedding X as an invariant set for a map *g* acting on the interval *I* which is the convex hull of the Cantor set X. We simply put ${g|}_{X}=\hat{f}$ and require $g\left(I_{e}\right)=I_{f(e)}$ by defining an affine map between images of endpoints, provided that two intervals $I_{e},I_{f(e)}$ are well defined. Finally, we define $g(I_{c})=I$ by sending endpoints of $I_{c}$ onto endpoints of *I* and defining *g* as an affine map between them. This way, we obtain a piecewise affine map with three pieces of monotonicity (see Figure 7).

Let *x* be the point in X⊂I induced by the point $z_{0}$ for map *f* from Section 3. Note that by the definition $\alpha_{\hat{f}}\left(x\right)\subset \alpha_{g}\left(x\right)$. We do not have equality of α-limit sets, however, because the image of $I_{c}$ by *g* is covering whole *I*. There is a point $z\in I_{c}$ such that g(z)=y for any y∈[0,1] and so each hole $I_{e}$ will contain pre-images of every point from $g^{-k}\left(x\right)$. Before we reveal what α(g(x)) exactly is, let us calculate the entropy of *g* and find support of the measure of maximal entropy, since these two problems are connected.

Clearly, $h_{top}\left(g\right)\geq h_{top}\left(f\right)$ since we may view $\hat{f}$ as a subsystem of *g*. However, the extension leading to *g* includes many new recurrent points originating from $I_{c}$. This set leads to numerous horseshoes defined by the sets $I_{q}$ for $q\in \cup_{k}f^{-k}\left(c\right)$. In fact, we have a kind of countable horseshoe compared to $\hat{f}$ (see Figure 7).

**Figure 8:**
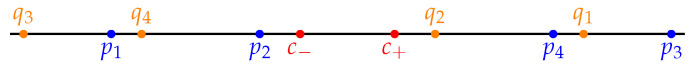
Relation between points $p_{i}$, $q_{i}$, and $c_{-},c_{+}$ for map *g*.

In the general case, to compute the entropy of map after blowup, we can use theory of Vere–Jones for countable Markov chains (e.g., see [35]), however revealing the direct structure of such a chain (infinite directed graph) is not easy. Fortunately, we have a nice Markov partition for the map *g*, see Figure 8, which is an immediate consequence of the structure in the map *f* (see Figure 3). Namely, points $p_{i},q_{i}$ do not enter the orbit of *c* and so were not blown up to construct $\hat{f}$. The only “new” point in Figure 3 are points $c_{-},c_{+}$ resulting from the interval $I_{c}$.

We obtain the following Markov diagram for *g*, where vertices are elements of partition, and symbols → schematically show vertices connected by arrows.

$$\begin{array}{ccc} \left[0,q_{3}\right] & \to & [p_{1},q_{4}];\\ {}[q_{3},p_{1}] & \to & [q_{4},p_{2}];\\ {}[p_{1},q_{4}] & \to & \left[p_{2},c_{-}\right],\left[c_{-},c_{+}\right],\left[c_{+},q_{2}\right];\\ {}[q_{4},p_{2}] & \to & \left[q_{2},p_{4}\right],\left[p_{4},q_{1}\right],\left[q_{1},p_{3}\right];\\ {}[p_{2},c_{-}] & \to & [p_{3},1];\\ {}[c_{-},c_{+}] & \to & \left[0,q_{3}\right],\left[q_{3},p_{1}\right],\left[p_{1},q_{4}\right],\left[q_{4},p_{2}\right],\left[p_{2},c_{-}\right],\left[c_{-},c_{+}\right],\\ & & \left[c_{+},q_{2}\right],\left[q_{2},p_{4}\right],\left[p_{4},q_{1}\right],\left[q_{1},p_{3}\right],\left[p_{3},1\right];\\ {}[c_{+},q_{2}] & \to & [0,q_{3}];\\ {}[q_{2},p_{4}] & \to & \left[q_{3},p_{1}\right],\left[p_{1},q_{4}\right],\left[q_{4},p_{2}\right];\\ {}[p_{4},q_{1}] & \to & \left[p_{2},c_{-}\right],\left[c_{-},c_{+}\right],\left[c_{+},q_{2}\right];\\ {}[q_{1},p_{3}] & \to & [q_{2},p_{4}];\\ {}[p_{3},1] & \to & [p_{4},q_{1}]. \end{array}$$

Again, calculating the leading eigenvalue λ of the associated matrix, we obtain that

$$h_{top}\left(g\right)=\log\lambda \approx \log\left(2.84005\right)\approx 1.50592.$$

By the variational principle, it means that our blow up procedure gave raise to a new ergodic measure ν, with entropy higher than previously observed in *f* in the measure of maximal entropy μ. Since ν is ergodic, it assigns full measure to the “holes” introduced along the backward trajectory of *c* (if a point enters X, it cannot leave it). Note, however, that *g* is topologically mixing as piecewise affine Markov map and X is nowhere dense. However, recent results show that for any *x* in the mixing interval map, $\alpha_{g}\left(x\right)$ contains all ω-limits sets of *g* (with only possible exception of α-limit sets of endpoints), e.g., see [36], Theorem 3.6. Therefore, $\alpha_{g}\left(x\right)=\left[0,1\right]$. This, among other things, means that the process of “filling holes” extended the considered α-limit set to a backward invariant set (which is no longer a proper subset).

## 7. When α-Limit Sets Are Invariant

We finish the paper with two simple observations ensuring when α(x) is backward invariant. This may be of independent interest, in particular as a tool in the construction of renormalization by Equation (3). By convention, we assume that f(c)=0.

**Proposition** **1.** *Let f be an expanding Lorenz map and let x be such that α(x)∩{f(0),f(1)}=∅. Then $f^{-1}\left(\alpha \left(x\right)\right)\subset \alpha \left(x\right)$.*

**Proof.**  Fix any $y\in f^{-1}\left(\alpha \left(x\right)\right)$ and note that by forward invariance of α(x), we have c∉α(x) since, by assumption, y∉{0,1}. Then, there is an open interval U=(a,b) such that y∈U and ${f|}_{U}$ is continuous and injective. If we denote z=f(y) and V=f(U), then *V* is an open interval and z∈α(x). By definition, there is a sequence $z_{n}\in {⋃}_{k\geq n}f^{-k}\left(\left\{x\right\}\right)$ such that $\lim_{n}z_{n}=z$ and, clearly, $z_{n}\in V$ for all *n* sufficiently large. However, for each *n*, there is a unique $y_{n}\in U$ such that $f\left(y_{n}\right)=z_{n}$. It is obvious that $y_{n}\in {⋃}_{k\geq n+1}f^{-k}\left(\left\{x\right\}\right)$ and passing to a convergent subsequence when necessary, we also must have $\lim_{n}y_{n}=y$ by continuity and the fact that *y* is the unique point in *U* such that f(y)=z. Indeed, y∈α(x), completing the proof. □

As we mentioned earlier, when an expanding Lorenz map *f* is topologically transitive, then $\cup_{n\geq 0}f^{-n}\left(\left\{x\right\}\right)$ is dense for every x∈[0,1] (e.g., see [23], Theorem 4.7) and as a result α(x)=[0,1]. This immediately leads to the following.

**Remark** **4.** *It may happen that 0,1∈α(x) but α(x) remains backward invariant. This shows that the condition in Proposition 1 is only a sufficient condition.*

**Proposition** **2.** *Let f be a unimodal interval map with the maximum at c and let x be such that f(c)∉α(x). Then $f^{-1}\left(\alpha \left(x\right)\right)\subset \alpha \left(x\right)$.*

**Proof.**  It is enough to see that if $y\in f^{-1}\left(\alpha \left(x\right)\right)$, then since y≠c, there is an open neighborhood y∈U such that ${f|}_{U}$ is injective and f(U) is an open set. It is mainly because *f* is monotone on *U*, 0 is a fixed point, and f(1)=0. Then, the rest of the proof follows the same lines as the proof of Proposition 1. □
